# Drug Overdose Deaths Among Medicaid Beneficiaries

**DOI:** 10.1001/jamahealthforum.2024.4365

**Published:** 2024-12-06

**Authors:** Tami L. Mark, Benjamin D. Huber

**Affiliations:** 1 RTI International, Washington, DC

## Abstract

**Importance:**

Medicaid programs have expanded coverage of substance use disorder treatment and undertaken many other initiatives to reduce drug overdoses among beneficiaries. However, to date, no information has been published that tracks overdose deaths among the Medicaid population.

**Objective:**

To determine the rate of drug overdose among Medicaid beneficiaries.

**Design, Setting, and Participants:**

In this cross-sectional study, US Centers for Medicare & Medicaid Services data from 2016 to 2020 that linked enrollment and demographic data from all Medicaid beneficiaries in the US with the US Centers for Disease Control and Prevention National Death Index were used to determine the rate of drug overdose death among Medicaid beneficiaries. The Medicaid population rates were compared with those of the total US population, overall and by age and sex.

**Exposure:**

Participation in the Medicaid program.

**Main Outcome:**

Death of a drug overdose.

**Results:**

In 2020, the drug overdose death rate among Medicaid beneficiaries was 54.6 per 100 000, a rate that was twice as high as the drug overdose rate among all US residents (27.9 per 100 000). In 2020, Medicaid beneficiaries comprised 25.0% of the US population but 48% of all overdose deaths (44 277 of 91 783). For each age and sex group older than 15 years, overdose deaths were higher for the Medicaid population than for the US population, with the greatest difference occurring among adults ages 45 to 64 years. From 2016 to 2020, Medicaid overdose deaths increased by 54%.

**Conclusions and Relevance:**

The results of this study suggest that more research is needed to understand why Medicaid beneficiaries have higher rates of drug overdoses than all US residents. Additionally, research is needed to understand how best to prevent overdoses among Medicaid beneficiaries. The federal government should support these efforts by routinely linking Medicaid claims and enrollment data to death records.

## Key Points

**Question:**

What is the rate of drug overdose among Medicaid beneficiaries?

**Findings:**

In this study, in 2020, the drug overdose death rate among Medicaid beneficiaries was 54.6 per 100 000 (44 277 of 91 783 overdose deaths), a rate that was twice as high as the drug overdose rate among all US residents (27.9 per 100 000). For each age and sex group older than 15 years, overdose deaths were higher for the Medicaid population than for the US population, with the greatest difference occurring among adults ages 45 to 64 years, and from 2016 to 2020, Medicaid overdose deaths increased by 54.%.

**Meaning:**

The results of this study suggest that drug overdose prevention interventions should be targeted toward Medicaid beneficiaries.

## Teaser

This cross-sectional study examines the rate of drug overdose among Medicaid beneficiaries.

## Introduction

In 2023, an unprecedented number of individuals in the US died of a drug overdose.^1^ The US overdose rate is much higher than in other high-income countries.^2^ Drug overdose deaths are a leading cause of injury deaths, and injury deaths are one of the top 5 causes of death in the US.^3,4^ In 2022, the federal government spent $39.3 billion on reducing drug use and overdose.^5^

To address the drug overdose epidemic, we must know what populations to prioritize. Population characteristics associated with overdose death include being male, young adulthood, having a disability, certain occupations, unstable housing, incarceration, unemployment, use of certain types of drugs (eg, fentanyl), and living in areas with high poverty and income inequality.^6,7,8,9,10,11^ Medications for opioid use disorders substantially reduce the risk of overdose death.^12^

In this study, we determined the rate of drug overdose deaths among Medicaid beneficiaries. Medicaid provides health coverage for millions of low-income adults and children and individuals with disabilities as well as nursing home coverage for low-income adults. To our knowledge, these are the first data published on the rate of overdose deaths among all Medicaid beneficiaries.

## Methods

### Data Sources and Description

The study was approved by the RTI institutional review board, which provided a waiver of informed consent. We followed the EQUATOR reporting guidelines. We used Medicaid beneficiary enrollment and demographic data from the US Centers for Medicare & Medicaid Service’s (CMS) Transformed Medicaid Statistical Information System (T-MSIS) files and the US Centers for Disease Control and Prevention (CDC) National Death Index (NDI) data for 2016 to 2020. For these years, CMS and CDC created a unique beneficiary identifier that allowed linkage of Medicaid enrollment and NDI data. The NDI data include the death date and the cause of death reported on the death certificates of decedents. The T-MSIS data without the NDI data contained an indicator of whether the Medicaid beneficiary died; however, the cause of death was not indicated, and the death indicator was based on state Medicaid enrollment information, which is not as accurate as the NDI. We identified the cause of death as a drug overdose using *International Statistical Classification of Diseases and Related Health Problems, Tenth Revision (ICD-10)* codes that the CDC uses (unintentional drug poisoning: X40-X44; suicide by drug poisoning: X60-X64; homicide by drug poisonings: X85; and drug poisoning of undetermined intent: Y10-Y14).^13^

### Overdose Death Rates

Using the T-MSIS–linked NDI data, we calculated the number of Medicaid beneficiaries who died each calendar year by age and sex group. The T-MSIS variables (beneficiary identifier, year age, and sex) were determined to be of high quality. To determine the denominator for the overdose death rate, we calculated the number of Medicaid beneficiaries enrolled each year adjusted by the number of months that they were enrolled (eg, a person enrolled for 6 months of the year would be counted as 0.5 person, whereas a person enrolled for 12 months of the year would be counted as 1 person). We divided the total number of overdose deaths each year by the month-weighted enrollment counts. The rates using the unweighted enrollment counts are reported in the eTable in Supplement 1 and do not differ meaningfully from the month adjusted rates. We compared the Medicaid overdose death rates by age group and sex with the overdose death rates for the whole US population as reported in the CDC’s WONDER online database.^14^

## Results

The 81 071 574 individuals enrolled in Medicaid are more likely to be female and younger than the US population (Table 1). Across all age groups, the Medicaid drug overdose death rate was 2 times the overall US drug overdose death rate (27.9 deaths vs 54.6 overdose deaths per 100 000) (Table 2). In 2020, Medicaid beneficiaries comprised 25% of the US population (81 071 574 of 329 484 123 individuals) but 48% of all overdose deaths (44 277 of 91 783 deaths). The overdose rate was low and equivalent for the age group younger than 15 years. For each age group older than 15 years, overdose deaths were higher for the Medicaid population than for the US population, with the greatest difference occurring among adults ages 45 to 64 years. For individuals ages 15 to 24 years, the rate was 1.2 times higher for Medicaid beneficiaries than the US population (16.7 vs 19.8 per 100 000); for ages 25 to 34 years, it was 1.9 times higher (47.3 vs 90.7 per 100 000); for ages 35 to 44 years, it was 2.4 times higher (53.9 vs 128.7 per 100 000); for ages 45 to 54 years, it was 3.2 times higher (46.9 vs 149.8 per 100 000); for ages 55 to 64 years, it was 3.3 times higher (37.3 vs 124.8 per 100 000); and for individuals ages 65 years and older, it was 2.8 times higher (9.4 vs 26.3 per 100 000). Examining the results by sex within age revealed that Medicaid beneficiaries had higher rates of overdose deaths than the total US population for every age group and sex combination except for female individuals younger than 15 years. Thus, the high rate of Medicaid overdose deaths cannot be explained by differences in the age or sex distribution of the Medicaid population compared with the US population.

**Table 1.** Age and Sex Distributions of US Residents and the Medicaid Population in 2020

Characteristic	US population, % (n = 329 484 123)^a^	Medicaid population, % (n = 81 071 574)^b^
Sex		
Female	50.8	55.4
Male	49.2	44.6
Age, y		
<15 y	18.3	32.8
15-24	12.9	16.5
25-34	14.0	13.3
35-44	12.8	10.5
45-54	12.3	8.1
55-64	12.9	8.7
≥65	16.9	10.1

^a^ Data from the US Centers for Disease Control and Prevention and National Center for Health Statistics via the National Vital Statistics System mortality data from 2018 to 2022 and WONDER Online Database (released in 2024). Data are from the Multiple Cause of Death Files from 2018 to 2022, as compiled from data provided by the 57 vital statistics jurisdictions through the Vital Statistics Cooperative Program.

^b^ Authors’ calculations based on US Centers for Medicare & Medicaid Service Transformed Medicaid Statistical Information System enrollment data and adjusted for months of enrollment.

**Table 2.** Overdose Deaths Among US Residents and Medicaid Beneficiaries by Selected Age and Sex Groups in 2020

Age group, y	US population, No.^a^			Medicaid population, No.^b^			Ratio^c^
	Residents	2020 Overdose deaths	Overdose deaths per 100 000	No. of full-year equivalent Medicaid beneficiaries^d^	2020 Overdose deaths	Overdose deaths per 100 000	
All ages	329 484 123	91 783	27.9	81 071 574	44 277	54.6	2.0
Female	167 227 921	28 069	16.8	44 940 026	15 484	34.5	2.1
Male	162 256 202	63 714	39.3	36 125 734	28 792	79.7	2.0
<15	60 293 426	247	0.4	26 589 431	116	0.4	1.0
Female	29 490 548	125	0.4	12 953 298	54	0.4	1.0
Male	30 802 876	122	0.4	13 632 833	62	0.5	1.3
15-24	42 555 684	7095	16.7	13 404 608	2654	19.8	1.2
Female	20 828 241	1990	9.6	7 277 654	952	13.1	1.4
Male	21 727 443	5105	23.5	6 126 849	1702	27.8	1.2
25-34	46 069 646	21 784	47.3	10 770 532	9769	90.7	1.9
Female	22 625 267	6141	27.1	6 924 416	3506	50.6	1.9
Male	23 444 379	15 643	66.7	3 846 060	6263	162.8	2.4
35-44	42 136 192	22 710	53.9	8 504 161	10 947	128.7	2.4
Female	21 090 324	6791	32.2	5 165 730	3887	75.2	2.3
Male	21 045 868	15 919	75.6	3 338 334	7060	211.5	2.8
45-54	40 366 133	18 919	46.9	6 551 617	9817	149.8	3.2
Female	20 441 441	6089	29.8	3 634 440	3458	95.1	3.2
Male	19 924 692	12 830	64.4	2 916 857	6358	218.0	3.4
55-64	42 403 677	15 819	37.3	7 063 411	8817	124.8	3.3
Female	21 914 243	5096	23.3	3 787 979	2932	77.4	3.3
Male	20 489 434	10 723	52.3	3 274 659	5885	179.7	3.4
≥65	55 659 365	5209	9.4	8 187 814	2157	26.3	2.8
Female	30 837 857	1837	5.6	5 196 509	695	13.4	2.4
Male	24 821 508	3372	13.6	2 990 143	1462	48.9	3.6

^a^ Data from the US Centers for Disease Control and Prevention and National Center for Health Statistics via the National Vital Statistics System mortality data from 2018 to 2022 and WONDER Online Database (released in 2024). Data are from the Multiple Cause of Death Files from 2018 to 2022, as compiled from data provided by the 57 vital statistics jurisdictions through the Vital Statistics Cooperative Program.

^b^ Authors’ calculations based on US Centers for Medicare & Medicaid Service Transformed Medicaid Statistical Information System enrollment data and adjusted for months of enrollment.

^c^ This percentage was calculated by dividing the Medicaid overdose rate by the all US overdose rate.

^d^ 5814 Transformed Medicaid Statistical Information System records (0.007% of the total number of records) were missing data on sex. All records are included in total estimates, while only records with data on sex are included in the female and male estimates.

From 2016 through 2020, the overdose rate among Medicaid beneficiaries increased by 54.2%, from 35.4 deaths to 54.6 deaths per 100 000 (Table 3). Data after 2020 for Medicaid beneficiaries are not available; therefore, we do not know if Medicaid overdose rates continued to rise through 2023 as they did for all overdose deaths.

**Table 3.** Overdose Deaths Among Medicaid Beneficiaries From 2016 to 2020

Year	No. of full-year–equivalent Medicaid beneficiaries^a^	Yearly overdose deaths among Medicaid beneficiaries	Overdose deaths per 100 000 Medicaid
2016	78 903 641	27 930	35.4
2017	79 073 193	32 591	41.2
2018	78 444 508	31 623	40.3
2019	77 053 359	33 067	42.9
2020	81 071 574	44 277	54.6
2021	90 764 383	NA	NA

Abbreviation: NA, not applicable.

^a^ Authors’ calculations based on US Centers for Medicare & Medicaid Service Transformed Medicaid Statistical Information System enrollment data and adjusted for months of enrollment.

## Discussion

The results of this study suggest that there is an urgent need to reduce drug overdose deaths among Medicaid beneficiaries. Although Congress, federal agencies, and states expanded Medicaid coverage of substance use disorder treatment, coverage gaps remain.^15^ Moreover, Medicaid laws and regulations largely only allow reimbursement for health care services delivered by Medicaid-participating health professionals to individual Medicaid beneficiaries. Thus, population-based harm reduction and primary prevention interventions, such as the distribution of fentanyl test strips and social marketing campaigns warning about the risk of fentanyl in the illicit drug supply, can only be paid for by more limited state and federal discretionary funding.

The Medicaid program can be viewed as a proxy for low-income individuals. The higher risk of drug use and overdose among Medicaid beneficiaries may be associated with the socioeconomic characteristics of Medicaid beneficiaries. Addressing the socioeconomic factors associated with substance use head on maybe more effective in the long term than focusing on preventing and treating substance use. However, more research is needed to understand whether programs and policies associated with improved socioeconomic characteristics are also associated with reduced drug use and associated harms.

This study was possible because CDC and CMS linked Medicaid data to NDI data. To our knowledge, CMS and CDC have no immediate plans to continue linking the data. Similarly, most states only link Medicaid and mortality data as a 1-time effort to answer a particular question. Policymakers and researchers need access to ongoing linked data to identify which Medicaid subpopulations are most at risk of drug overdoses and evaluate interventions to reduce drug overdose deaths.

### Limitations

This study did not account for deaths that may have been a consequence of drug use, such as infections, cardiac arrest, injuries, and exposure. Furthermore, to our knowledge, data do not exist to compare Medicaid with the non-Medicaid population. Therefore, we were only able to compare Medicare with the whole US population, which diluted the differences. Finally, the goal of this study was not to unpack why Medicaid is associated with overdose deaths, such as whether it is a proxy for social determinants of health or reflects drug patterns, or treatment receipt. This type of further research is clearly needed.

## Conclusions

This study suggests that drug overdose deaths remain a leading cause of death in the US despite years of investment to try to address the opioid epidemic. Medicaid beneficiaries are at high risk of drug overdoses. Federal and states agencies should invest in timely and accessible linked mortality and Medicaid data to better understand and target interventions toward the populations at highest risk.

## Notes

Supplement 1. eAppendix. eTable. Overdose deaths among US residents and all Medicaid beneficiaries not weighted by Month, by selected age groups, 2020

Supplement 2. Data sharing statement
