# Supplementary material for: Drug Overdose Deaths Among Medicaid Beneficiaries
Source: JAMA Health Forum. 2024 Dec 6;5(12):e244365. doi: 10.1001/jamahealthforum.2024.4365 (PMC11624576; doi:10.1001/jamahealthforum.2024.4365)
Supplement: Supplement 1. — eAppendix. eTable. Overdose deaths among US residents and all Medicaid beneficiaries not weighted by Month, by selected age groups, 2020 [file jamahealthforum-e244365-s001.pdf]

Supplemental Online Content

Mark TL, Huber BD. Drug overdose deaths among Medicaid beneficiaries. *JAMA Health Forum*. 2024;5(12):e244365. doi:10.1001/jamahealthforum.2024.4365

eAppendix.

eTable. Overdose deaths among US residents and all Medicaid beneficiaries not weighted by Month, by selected age groups, 2020

This supplemental material has been provided by the authors to give readers additional information about their work.

© 2024 Mark TL et al. *JAMA Health Forum.*

eAPPENDIX

Statistics in eTabel 1. are presented for all beneficiaries ever enrolled in Medicaid in a year. Where the enrollment-adjusted rates in the main study weight individuals by the number of months enrolled in Medicaid in the year, the measurements in this appendix treat all enrollees, whether enrolled for one month or twelve, as a single beneficiary. Because of this method, the number of overall beneficiaries appears somewhat larger than the numbers reported above, and because the counts over overdose deaths are the same, the overdose mortality rates among Medicaid beneficiaries appear somewhat smaller. The rates and counts of residents and overdose deaths for the entire U.S. population are identical to those reported above, and are presented here for comparison.

© 2024 Mark TL et al. *JAMA Health Forum.*

eTable. Overdose deaths among US residents and all Medicaid beneficiaries not weighted by Month, by selected age groups, 2020

	U.S. Population[a]			Medicaid Population[b]			Ratio[c]
**Age Group (years)**	**Residents by age**	**2020 Overdose deaths**	**Overdose deaths per 100,000**	**Number of Medicaid Beneficiaries**[d]	**2020 Overdose deaths**	**Overdose deaths per 100,000**	
**All-Ages**	329,484,123	91,783	27.9	91,086,541	44,277	48.6	1.7
**Male**	162,256,202	63,714	39.3	40,562,055	28,792	71.0	1.8
**Female**	167,227,921	28,069	16.8	50,518,281	15,484	30.7	1.8
**<15**	60,293,426	247	0.4	28,792,703	116	0.4	1.0
Male	30,802,876	122	0.4	14,754,397	62	0.4	1.0
Female	29,490,548	125	0.4	14,034,878	54	0.4	1.0
**15-24**	42,555,684	7,095	16.7	15,209,412	2,654	17.4	1.0
Male	21,727,443	5,105	23.5	6,861,101	1,702	24.8	1.1
Female	20,828,241	1,990	9.6	8,348,175	952	11.4	1.2
**25-34**	46,069,646	21,784	47.3	12,807,589	9,769	76.3	1.6
Male	23,444,379	15,643	66.7	4,633,357	6,263	135.2	2.0
Female	22,625,267	6,141	27.1	8,174,145	3,506	42.9	1.6
**35-44**	42,136,192	22,710	53.9	9,812,040	10,947	111.6	2.1
Male	21,045,868	15,919	75.6	3,905,256	7,060	180.8	2.4
Female	21,090,324	6,791	32.2	5,906,667	3,887	65.8	2.0
**45-54**	40,366,133	18,919	46.9	7,473,459	9,817	131.4	2.8
Male	19,924,692	12,830	64.4	3,360,067	6,358	189.2	2.9
Female	20,441,441	6,089	29.8	4,113,042	3,458	84.1	2.8
**55-64**	42,403,677	15,819	37.3	7,930,825	8,817	111.2	3.0
Male	20,489,434	10,723	52.3	3,704,721	5,885	158.9	3.0
Female	21,914,243	5,096	23.3	4,225,280	2,932	69.4	3.0
**65+**	55,659,365	5,209	9.4	9,060,513	2,157	23.8	2.5
Male	24,821,508	3,372	13.6	3,343,156	1,462	43.7	3.2
Female	30,837,857	1,837	5.6	5,716,094	695	12.2	2.2

Sources:

a. Centers for Disease Control and Prevention, National Center for Health Statistics. National Vital Statistics System, Mortality 2018-2022 on CDC WONDER Online Database, released in 2024. Data are from the Multiple Cause of Death Files, 2018-2022, as compiled from data provided by the 57 vital statistics jurisdictions through the Vital Statistics Cooperative Program. Accessed at http://wonder.cdc.gov/ucd-icd10-expanded.html

b. Author's calculations based on CMS T-MSIS enrollment data and adjusted for months of enrollment.

c. This percentage is calculated by dividing the Medicaid overdose rate by the all US overdose rate.

d. Some T-MSIS records are missing data on sex, all records are included in total estimates, while only records with data on sex are included in the male and female estimates.

© 2024 Mark TL et al. *JAMA Health Forum.*
