# Supplementary material for: Drug Overdose Deaths Among Medicaid Beneficiaries
Source: JAMA Health Forum. 2024 Dec 6;5(12):e244365. doi: 10.1001/jamahealthforum.2024.4365 (PMC11624576; doi:10.1001/jamahealthforum.2024.4365)
Supplement: Supplement 2. — Data sharing statement [file jamahealthforum-e244365-s002.pdf]

Data Sharing Statement

Mark. Drug Overdose Deaths Among Medicaid Beneficiaries. *JAMA Health Forum*. Published December 06, 2024. doi:10.1001/jamahealthforum.2024.4365

Data
Data available: No

Additional Information
Explanation for why data not available: the individual data is protected by HIPAA and sharing would violate our Data Use Agreement with CMS.
